# Editorial: Diet behavior and heart health

**DOI:** 10.3389/fnut.2024.1376712

**Published:** 2024-02-27

**Authors:** Jiming Kang, Liliang Yu, Hongtao Tie, Cesar Reis, Yong Zhao

**Affiliations:** ^1^Chongqing Three Gorges Medical College Chongqing, Chongqing, China; ^2^Department of Cardiothoracic Surgery, First Affiliated Hospital of Chongqing Medical University, Chongqing, China; ^3^United States Department of Veterans Affairs, VA Loma Linda Healthcare System, Veterans Health Administration, Loma Linda, CA, United States; ^4^School of public health, Chongqing Medical University, Chongqing, China

**Keywords:** diet behavior, heart health, cardiovascular diseases, dietary pattern, food component

The association between diet and health has been identified, and a healthy diet can prevent various cardiovascular diseases (CVDs) (1). Dietary behaviors and patterns are changing, especially in developing countries with the ever-increasing amount of processed food and changing lifestyles, people eat more foods rich in energy, fat, free sugars, and salt, but not enough in fruits, vegetables, and other dietary fibers (2).

CVDs, including coronary heart disease, heart failure, stroke, and hypertension, are the leading cause of mortality worldwide. Poor dietary behaviors and patterns are risk factors for the continuing increase of CVDs incidence, accounting for more than 11 million deaths (3, 4). Although there are accumulating manuscripts investigating the relationship between dietary behaviors and patterns and CVDs, more high-quality evidence to support the improvement of cardiovascular health through dietary behavior is needed.

This Research Topic aims to investigate the relationship between diet behavior, including various dietary patterns, adverse dietary behaviors, and trace elements in food, and heart health. A total of 20 studies have been published on this Research Topic. The researchers focused on the effects of different eating patterns, several different food groups, and the eating behavior of different populations on heart health.

Poor diet quality is closely associated with CVD morbidity and mortality (5). The American Heart Association (AHA) and many other researchers have focused on the impact of dietary patterns on heart health, such as the DASH dietary pattern, the Mediterranean dietary pattern (MED), and the plant-based food dietary pattern (6–8). Notably, in a cross-sectional and longitudinal study, Dou et al. revealed for the first time the value of the Mediterranean-Dietary Approaches to Stop Hypertension for neurodegenerative delay (MIND) dietary pattern, a promising dietary pattern designed from most of the ingredients in the MED and DASH diets, in the primary and secondary prevention of hypertension, suggesting the MIND diet as a novel anti-hypertensive dietary pattern. In addition, in a prospective cohort study based on the 2007–2014 National Health and Nutrition Examination Survey, Zhang Y. et al. showed that adherence to higher intake of green vegetables and legumes, vegetables, total protein foods, seafood and plant protein, unsaturated fatty acids, and moderate intake of empty calories was associated with lower all-cause mortality from hypertension. Wang et al. show that diets low in whole grains, low in legumes and high in sodium are the three main dietary factors that increase the risk of ischemic heart disease burden. These studies will promote dietary patterns that affect heart health-related research.

Five publications investigated the effects of foods and their various components on heart health. Yang X. et al. conducted a systematic review and dose-response meta-analysis showing that drinking more green tea can reduce the risk of coronary heart disease, but drinking more than 4–6 cups of black tea per day may increase the risk of coronary heart disease, which provides new insights into the relationship between tea consumption and its preventive effect on coronary heart disease. Chen et al. have shown that moderate caffeine intake can reduce all-cause mortality and cardiovascular mortality in elderly patients with hypertension. Huang Y. et al. conducted a retrospective cohort study showing that drinking very low-mineral water may increase homocysteine level and oxidative stress, worsen lipid profile, and threaten the cardiovascular system in children, while reducing 1,25, (OH)2D3, and disordering of calcium metabolism might play important roles. Huang J. et al. conducted a Mendelian randomized study showing that socioeconomic status, which was closely associated with other eating habits and lifestyle, may affect the association between vegetable intake and ischemic cardio-cerebral vascular diseases. Yang Y. et al. showed that decreasing the intake of edible oil n-6/n-3 ratio can improve blood lipids and quality of life.

In previous studies, few studies have been conducted on the effects of dietary behaviors on heart health in different populations. Notably, in this Research Topic, four publications have respectively studied adults, people in rural areas, people with sleep disorders, and only children, which will provide new ideas for the study of the effect of diet on heart health. Rikhtehgaran et al. conducted a prospective population cohort study showing that adults in the unhealthy diet group had twice the risk of developing CVD. Zhang J. et al. showed that in patients with sleep disorders, a higher intake of red and orange vegetables, starchy vegetables, and fermented dairy products in the morning, and a higher intake of milk and eggs in the evening, were associated with a lower risk of cardiovascular disease. Dang et al. show that single children with a poor lifestyle are significantly associated with the risk of developing cardio-metabolic risk factors, and that increasing family size (number of siblings) or establishing a good lifestyle may partially offset this risk. Hou et al. show that a healthy diet, such as high consumption of vegetables and seafood, as well as foods rich in selenium, may help prevent and control hypertension in Keshan endemic areas and other rural areas in China.

All in all, this Research Topic provides new ideas and insights into the influence of dietary behavior on heart health, especially for different populations and diversified eating patterns. Although a large number of studies have been carried out in this field, due to the diversity of food types, the complexity of food collocation, and the differences between different populations, the impact of dietary behavior on heart health needs more in-depth and extensive research. Future research should focus on the dietary behaviors of different regional cultures and different populations, the effects of processed foods, and diverse dietary patterns on heart health.

## Author contributions

JK: Funding acquisition, Resources, Writing—review & editing. LY: Writing—original draft, Writing—review & editing. HT: Writing—original draft, Writing—review & editing. CR: Writing—original draft, Writing—review & editing. YZ: Funding acquisition, Methodology, Writing—original draft, Writing—review & editing.
